# Enhancing Pulmonary Disease Prediction Using Large Language Models With Feature Summarization and Hybrid Retrieval-Augmented Generation: Multicenter Methodological Study Based on Radiology Report

**DOI:** 10.2196/72638

**Published:** 2025-06-11

**Authors:** Ronghao Li, Shuai Mao, Congmin Zhu, Yingliang Yang, Chunting Tan, Li Li, Xiangdong Mu, Honglei Liu, Yuqing Yang

**Affiliations:** 1 School of Biomedical Engineering, Capital Medical University, No. 10, Xitoutiao, You An Men, Fengtai District, Beijing, 100069, China, 86 010-83911542; 2 State Key Laboratory of Networking and Switching Technology, Beijing University of Posts and Telecommunications, Beijing, China; 3 Department of Respiratory Medicine, Beijing Friendship Hospital, Capital Medical University, Beijing, China; 4 Beijing Respiratory and Critical Care Medicine Department, Tsinghua Changgung Hospital, School of Clinical Medicine, Tsinghua University, Beijing, China

**Keywords:** retrieval-augmented generation, large language models, prompt engineering, pulmonary disease prediction, RAG, LLM

## Abstract

**Background:**

The rapid advancements in natural language processing, particularly the development of large language models (LLMs), have opened new avenues for managing complex clinical text data. However, the inherent complexity and specificity of medical texts present significant challenges for the practical application of prompt engineering in diagnostic tasks.

**Objective:**

This paper explores LLMs with new prompt engineering technology to enhance model interpretability and improve the prediction performance of pulmonary disease based on a traditional deep learning model.

**Methods:**

A retrospective dataset including 2965 chest CT radiology reports was constructed. The reports were from 4 cohorts, namely, healthy individuals and patients with pulmonary tuberculosis, lung cancer, and pneumonia. Then, a novel prompt engineering strategy that integrates feature summarization (F-Sum), chain of thought (CoT) reasoning, and a hybrid retrieval-augmented generation (RAG) framework was proposed. A feature summarization approach, leveraging term frequency–inverse document frequency (TF-IDF) and K-means clustering, was used to extract and distill key radiological findings related to 3 diseases. Simultaneously, the hybrid RAG framework combined dense and sparse vector representations to enhance LLMs’ comprehension of disease-related text. In total, 3 state-of-the-art LLMs, GLM-4-Plus, GLM-4-air (Zhipu AI), and GPT-4o (OpenAI), were integrated with the prompt strategy to evaluate the efficiency in recognizing pneumonia, tuberculosis, and lung cancer. The traditional deep learning model, BERT (Bidirectional Encoder Representations from Transformers), was also compared to assess the superiority of LLMs. Finally, the proposed method was tested on an external validation dataset consisted of 343 chest computed tomography (CT) report from another hospital.

**Results:**

Compared with BERT-based prediction model and various other prompt engineering techniques, our method with GLM-4-Plus achieved the best performance on test dataset, attaining an *F*_1_-score of 0.89 and accuracy of 0.89. On the external validation dataset, *F*_1_-score (0.86) and accuracy (0.92) of the proposed method with GPT-4o were the highest. Compared to the popular strategy with manually selected typical samples (few-shot) and CoT designed by doctors (*F*_1_-score=0.83 and accuracy=0.83), the proposed method that summarized disease characteristics (F-Sum) based on LLM and automatically generated CoT performed better (*F*_1_-score=0.89 and accuracy=0.90). Although the BERT-based model got similar results on the test dataset (*F*_1_-score=0.85 and accuracy=0.88), its predictive performance significantly decreased on the external validation set (*F*_1_-score=0.48 and accuracy=0.78).

**Conclusions:**

These findings highlight the potential of LLMs to revolutionize pulmonary disease prediction, particularly in resource-constrained settings, by surpassing traditional models in both accuracy and flexibility. The proposed prompt engineering strategy not only improves predictive performance but also enhances the adaptability of LLMs in complex medical contexts, offering a promising tool for advancing disease diagnosis and clinical decision-making.

## Introduction

The rapid advancement of artificial intelligence (AI) has significantly driven the development of medical auxiliary diagnosis systems. Algorithms based on electronic health records (EHRs), using machine learning and deep learning techniques, have demonstrated considerable potential in predicting various clinical conditions and outcomes, such as lung cancer [1,2], breast cancer [3], and type 1 diabetes [4].

The Transformer architecture [5] has effectively addressed the challenge of capturing long-range dependencies within sentences, significantly enhancing models’ abilities to understand contextual relationships. Consequently, Transformer-based models, such as Bidirectional Encoder Representations from Transformers (BERT) [6], have become the dominant approach for text representation learning. More recently, the advent of large language models (LLMs), including ChatGPT (OpenAI), BARD (Google), and LLaMA (Meta AI), has provided researchers with novel tools for analyzing complex medical data [7-10], particularly clinical text. LLMs have shown excellent performance in tasks such as information extraction [11-14], text summarization [15,16], and text generation [17]. For example, BiomedGPT, the first open-source and lightweight visual LLM, was designed to perform various biomedical tasks [18], such as multimodal-based disease examination, auxiliary diagnosis, and information extraction. However, while benchmarks for general natural language processing (NLP) tasks exist, their efficacy in auxiliary diagnosis tasks remains underexplored [19-21]. These findings underscore the need for targeted design and optimization of LLMs to address the unique demands of specific medical tasks. One critical issue is that most general-purpose LLMs are trained on open-domain text and lack sufficient domain-specific adaptation, making it difficult for them to interpret the highly specialized, ambiguous, and context-dependent language used in clinical texts. Furthermore, commonly used prompt strategies, such as few-shot learning or manually designed reasoning chains (eg, chain of thought [CoT]), often rely on handcrafted examples and static templates, which are difficult to scale and generalize across diverse disease types and institutions. As a result, such models often suffer from limited robustness, reduced interpretability, and suboptimal diagnostic performance in real-world clinical settings.

Pulmonary diseases, including lung cancer, pneumonia, and tuberculosis, remain among the leading causes of morbidity and mortality worldwide. These 3 diseases were selected due to their high clinical prevalence, significant public health burden, and distinct imaging and pathological characteristics, which provide a robust foundation for evaluating the performance of AI-based diagnostic tools. In addition, their associated radiology reports often contain intricate medical terminology, ambiguous symptom descriptions, and heterogeneous clinical information, posing challenges for accurate analysis and decision-making. Therefore, developing high-quality NLP methods targeting these conditions is crucial to improve disease prediction, facilitate early diagnosis, and enhance prognosis modeling. In recent years, AI-based approaches, particularly Transformer-based architectures, have shown significant potential in pulmonary disease diagnosis by extracting informative features from diverse types of clinical data, such as radiology reports, pathology results, and electronic health records (EHRs) [2,22,23]. For instance, Shao et al [24] combined BERT and other deep learning models with the Swin Transformer to distinguish between bacterial, fungal, and viral pneumonia, as well as tuberculosis, demonstrating the effectiveness of hybrid architectures in multi-class classification tasks. Cho et al [25] used pathology reports and gold standard to generate prompt-response pairs for training and then applied GLMs to extract information for staging from pathology reports.

Despite these advancements, existing methods often struggle to capture the nuanced clinical context embedded in free-text narratives while maintaining interpretability in decision-making processes. In medical-assisted diagnosis, the predictive performance of traditional fine-tuning-based deep learning models is often constrained by the limited availability of single-center data, imbalanced data distributions, and variations in reporting standards across multiple institutions. In addition, fine-tuning and optimizing these models require extensive expertise from AI specialists, which to some extent hinders their widespread clinical adoption.

In this study, we aimed to address the practical limitations of existing large language models LLMs in real-world diagnostic scenarios, particularly their insufficient domain-specific adaptation, challenges in interpreting complex clinical language, and reliance on rigid and handcrafted prompting strategies. To overcome these issues and facilitate accurate pulmonary disease diagnosis, we developed a novel prompt engineering strategy based on LLMs, combined with unsupervised learning technique, to explore new effective diagnostic assistance method. Inspired by the clinical reasoning process, our approach integrates automatic feature summarization, structured CoT reasoning, and a hybrid retrieval-augmented generation (RAG) mechanism. Together, these components enhance the ability of LLMs to understand and reason over radiology report texts, enabling accurate prediction of diseases such as pneumonia, tuberculosis, and lung cancer. We systematically compared our method with multiple prompting strategies and validated its performance using a multi-center dataset. Furthermore, comparing to the traditional fine-tuned BERT model, we demonstrated the superior predictive performance and generalizability of our approach. The proposed new method can achieve favorable prediction results without the need for complex fine-tuning process, making it convenient for integration into existing clinical workflows to assist physicians in diagnosing diseases.

## Methods

### Dataset Description

This study used 2965 chest computed tomography (CT) radiology reports collected from Beijing Friendship Hospital in Beijing, China, spanning the years 2012 to 2018. The dataset comprised 367 reports from healthy individuals, 534 from patients with pulmonary tuberculosis, 553 from patients with lung cancer, and 1511 from patients with pneumonia. The original texts of reports were extracted from medical information system and labels for the image findings were annotated by 2 respiratory physicians based on the report texts. Personal privacy information in the reports and reports with vague descriptions, diagnostic discrepancies, and text extraction errors were removed. Any discrepancies between their annotations were resolved through consensus with a senior physician to ensure accuracy. Each radiology report contained two key sections: image findings and labels. The dataset was then used to develop a multiclassification model for pulmonary disease prediction. Reports were further divided into a training set and a test set in an 8:2 ratio.

In addition, an external validation dataset of 343 chest CT radiology reports was collected from Beijing Tsinghua Changgung Hospital. This dataset included 31 reports from healthy individuals, 23 reports from patients with pulmonary tuberculosis, 93 reports from patients with lung cancer, and 196 reports from patients with pneumonia.

### LLM-Based Classification Model

#### Overview

**Figure 1.**
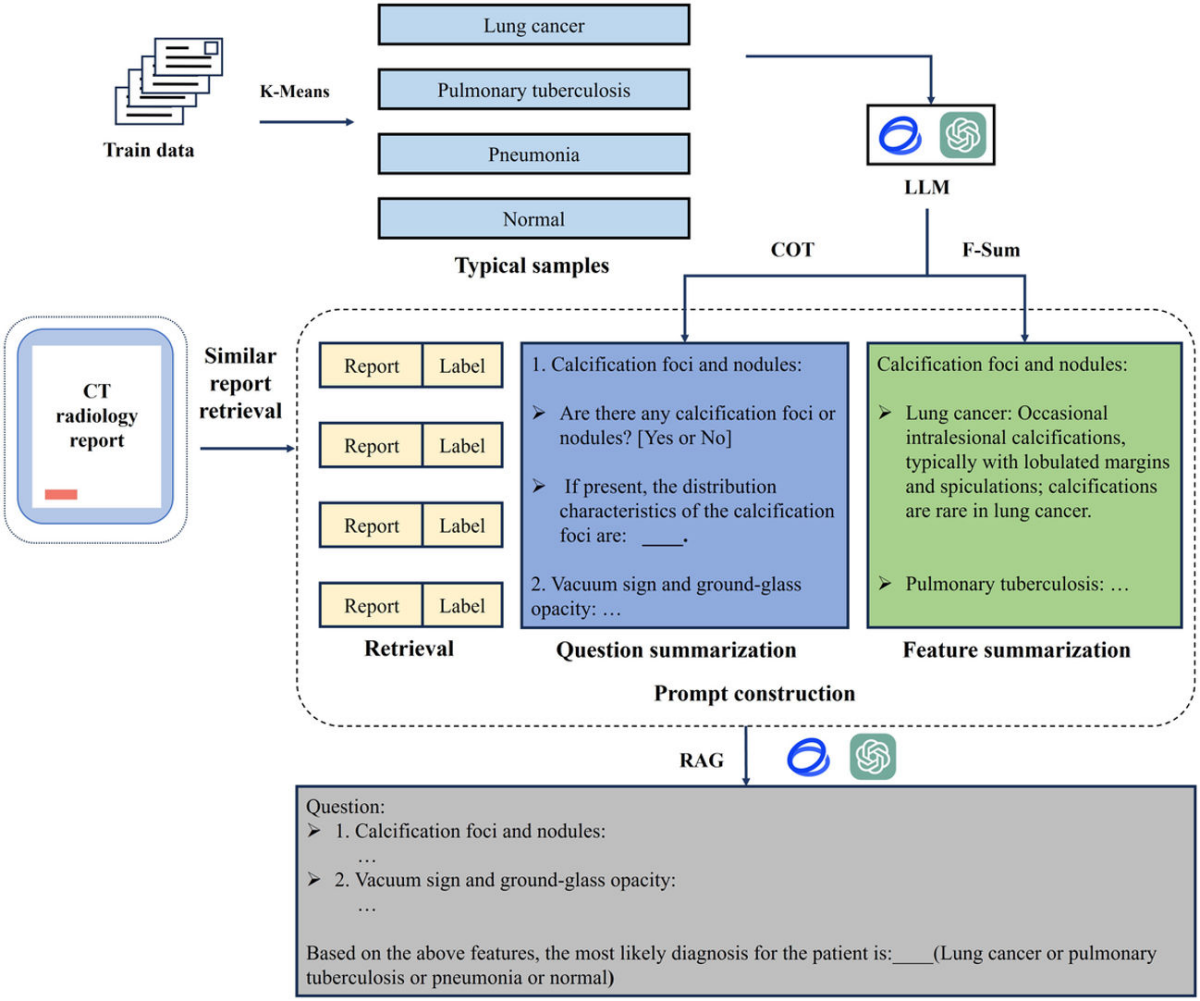
Workflow of the large language model (LLM)–based classification model. COT: chain of thought; CT: computed tomography; F-Sum: feature summarization; RAG: retrieval-augmented generation.

We improved the prompt engineering technology and developed a novel LLM-based classification framework by integrating 3 key components: clustering-based feature summarization, CoT, and hybrid RAG. Initially, term frequency–inverse document frequency (TF-IDF) and K-means clustering techniques are employed to extract representative radiological reports, which are subsequently summarized by LLMs. Based on summarized diseases features, LLM is used to generate CoT-based diagnostic inquiries to enhance structured reasoning. Then a hybrid RAG framework is implemented with both dense and sparse vector representations to retrieve similar reports. Finally, combined information from feature summarization, CoT reasoning, and hybrid retrieval was included in the prompt for LLMs to perform pulmonary disease prediction. This framework is designed to emulate a physician’s cognitive process in learning and diagnosing diseases, thereby enhancing predictive accuracy by identifying characteristic patterns in diseased populations and analyzing similarities with comparable cases. Figure 1 illustrates the workflow of the proposed prompt engineering strategy.

To enhance the understanding of complex medical terminology and radiological features, the workflow begins with feature summarization, where representative imaging findings for each disease are obtained using the TF-IDF and K-means clustering method. These findings are further analyzed by the LLM to extract disease-specific features and rank their importance. Then the summarized features are used by the LLM to generates diagnostic questions to construct a logical reasoning pathway. During the prediction phase, the workflow retrieves similar imaging reports via a hybrid RAG framework to refine the LLM’s understanding of disease patterns, ultimately generating comprehensive and precise results for disease prediction.

#### Feature Summarization (F-Sum)

Feature summarization (F-Sum) serves as the foundational step for learning disease characteristics by analyzing radiology text and summarizing key features. To facilitate interpretable and efficient feature extraction from radiology report texts, we adopted the TF-IDF method, which assigns importance scores to medical terms based on their frequency and specificity. This enables the selection of clinically relevant descriptors without requiring extensive labeled data. For clustering, we used K-means due to its simplicity and effectiveness in grouping similar reports, which supports coherent feature summarization.

First radiology report text is transformed into a numerical feature matrix using TF-IDF and subsequently normalized. The top 5000 features with the highest TF-IDF weights are retained. For each disease category, the K-means algorithm is applied, with the maximum number of clusters set to 20 or the total sample size of that category. Representative samples are proportionally selected from each cluster based on cluster size to ensure diversity. The samples closest to the cluster centroid are chosen to maintain category representativeness and balance. This step is predeveloped before analyzing the text with LLMs.

**Figure 2.**
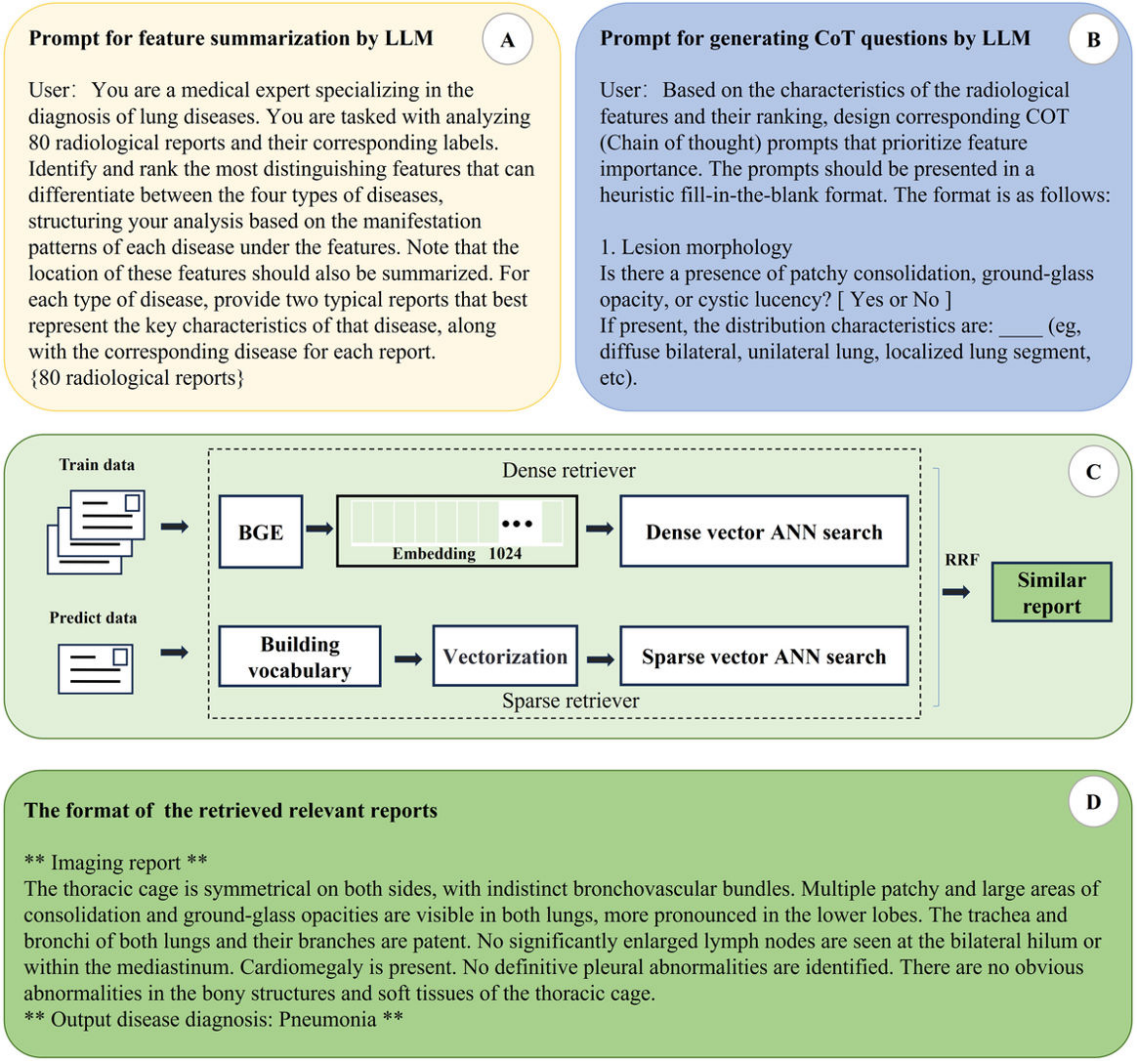
Detailed strategy diagrams for each section. A. The prompt for feature summarization using the large language model (LLM). B. The prompt for generating chains of thought (CoT) questions with LLM. C. The hybrid retrieval-augmented generation (RAG) process for retrieving similar reports based on both dense and sparse vector representations. D. The format of similar reports retrieved by RAG in the final prompt for LLMs. ANN: approximate nearest neighbor search; BGE: BAAI General Embedding; RRF: relevance-weighted rank fusion.

Then the representative samples derived from clustering are analyzed by the LLM to summarize key disease-specific features (see Figure 2A). The LLM ranks these features by importance, allowing the model to prioritize critical characteristics in the diagnostic decision-making process. The tasks of feature summarization and chain of thought (CoT) analysis were executed using the GLM4-Plus model.

#### CoT Reasoning

The CoT reasoning component guides the model through a step-by-step diagnostic process, enhancing interpretability and replicating clinical reasoning. Based on the representative imaging findings from clustering, the LLM generates diagnostic questions designed to emulate a physician’s thought process (see Figure 2B). These questions support the model in systematically deriving disease predictions by simulating a logical diagnostic workflow.

#### Hybrid RAG

The RAG technique enhances the LLM’s generative capabilities by incorporating external knowledge through retrieval. In the disease classification task, RAG enables the model to extract insights from similar cases, improving diagnostic precision.

We implemented a hybrid retrieval system that integrates dense and sparse vector representations (see Figure 2C), using the Milvus database. Dense vectors were generated using the pretrained language model BGE-large-zh-v1.5. Average pooling was applied to create fixed-length embeddings. A custom vocabulary was built based on the imaging reports. Texts were converted into a term frequency matrix to generate sparse vector representation.

During retrieval, similarity queries were performed using L2 Euclidean distance, and the top 50 results were identified through nearest neighbor search. The results from both retrieval methods were then reranked using the Relevance-Weighted Rank Fusion (RRF) Ranker, merging them into a single retrieval result table to balance the contributions of different retrieval approaches,


$$RRF\left(d\right)=\sum_{r\in R}\frac{1}{k+r\left(d\right)}$$

where *k*=60, and *r* represents the similarity score in a specific retriever method. The top 4 most relevant imaging reports and disease labels were retained for subsequent predictions. Retrieved reports were formatted into prompts as shown in Figure 2D, enabling the LLM to leverage similar case knowledge for more accurate predictions.

### Mode Training and Evaluation

#### LLM Selection

We used novel prompt engineering strategies tailored for 3 prominent LLMs: GLM4-plus, GLM4-air, and GPT-4o. GLM4-plus and GLM4-air, developed by Zhipu AI, are general-purpose Transformer-based models optimized for Chinese NLP tasks, excelling in text comprehension and generation. GPT-4o, launched by OpenAI, inherits the advanced generative capabilities of the GPT series and introduces significant enhancements in multimodal processing and algorithm efficiency.

To compare our approach with traditional deep learning approaches, we selected BERT as the baseline. BERT is a pretrained transformer-based language representation model that has demonstrated exceptional performance in a range of NLP tasks, including text classification, sentiment analysis, and named entity recognition.

#### Comparisons Among Various LLM Prompt Engineering Strategies

As shown in Table 1, we evaluated multiple different prompt engineering strategies by combining few-shot, F-Sum, CoT, and RAG, across the 3 LLMs: GLM4-air, GLM4-plus, and GPT-4o. Few-shot learning improves LLM performance by exposing the model to a limited number of examples relevant to the target task. For the few-shot, fixed samples for each disease category were provided to guide the model’s learning process before performing prediction tasks. In addition, we compared these strategies with manually designed prompt, denotated as the “Few-shot + CoT + RAG (manual prompt).” In this approach, both the few-shot learning samples and the CoT questions were designed through an expert-driven process, involving direct communication with medical specialists. This methodology emulates expert diagnostic procedures by incorporating critical features for distinguishing between diseases and formulating CoT questions that promote a deeper understanding and differentiation of medical conditions. The comparative analysis of predictive performance across these strategies provides a more comprehensive evaluation of the F-Sum + CoT + RAG approach, particularly highlighting its advantages in handling complex medical tasks.

**Table 1.** Performance comparison of different prompt engineering strategies based on large language models (LLMs).

LLM prompt engineering strategy	GLM4-air	GLM4-Plus	GPT4o
Few-shot + CoT^a^	—^b^	+^c^	—
F-Sum + ^d^Few-shot	+	+	+
F-Sum + RAG^e^	+	—	—
F-Sum + Few-shot + CoT	+	+	+
F-Sum + CoT + RAG	+	+	+
F-Sum + CoT + RAG (BERT^f^ embed cluster)	+	—	—
Few-shot + CoT + RAG (manual prompt)	+	—	—

a CoT: chain of thought.

b Not applicable.

c Denotes the use of the respective method.

d F-Sum: feature summarization.

e RAG: retrieval-augmented generation.

f BERT: Bidirectional Encoder Representations from Transformers.

#### Comparison With BERT-Based Model

To ensure a fair comparison, the BERT-based model was trained and tested using the same datasets as the LLMs. Text data was encoded into vector representations using BERT-base-Chinese as the backbone. The model consisted of 12 stacked BERT layers, each with 768 filters, and produced an embedding dimension of 768 after the BertPooler layer. A linear classification layer was added on top of the word vectors, and the Softmax function was used for predicting the categories of pulmonary diseases (Figure S1 in Multimedia Appendix 1). During training, the AdamW optimizer was used with a learning rate of 1×10^–5^. The model was trained over 30 epochs with a batch size of 16. The maximum input length was set to 510 tokens to accommodate longer sequences.

The experiments were conducted on a single NVIDIA A6000 GPU (48 GB memory) with Python (version 3.8.5; Python Software Foundation), PyTorch 2.0.0+cu117, CUDA 11.7, and Ubuntu 22.04.1.

#### Evaluation Metrics

We used multiple evaluation metrics, including accuracy, precision, recall, and *F*_1_-score, to comprehensively assess model performance. For multiclassification tasks, we used the macro-average approach, which calculates the metrics (precision, recall, and *F*_1_-score) for each class individually and then computes their arithmetic mean across all classes.

### Ethical Considerations

The study was approved by the Human Research Ethics Committees of Beijing Friendship Hospital (2023-P2-031-01) and Tsinghua Changgung Hospital (23694-4-01) with an exemption from informed consent from. All data used were anonymized to ensure participant privacy.

## Results

The performance of lung disease prediction using different prompt engineering strategies with LLMs and BERT is summarized in Table 2. On the test dataset, the F-Sum + CoT + RAG strategy achieved the highest results among all LLM-based methods. GLM-4-air achieved an *F*_1_-score of 0.89 and accuracy of 0.90, while GLM-4-plus reached an *F*_1_-score of 0.89 and accuracy of 0.89. Notably, the F-Sum + CoT + RAG strategy demonstrated a balanced performance, achieving the highest recall (0.92) with GLM-4-air and the highest precision (0.88) with GPT-4o. These results indicate that the F-Sum + CoT + RAG strategy enhances predictive accuracy and achieves a good trade-off between recall and precision.

On the external validation dataset, GPT-4o with F-Sum + CoT + RAG outperformed other models, achieving the highest *F*_1_-score (0.86) and accuracy (0.92). It also maintained high recall (0.91) and precision (0.86), showcasing its strong generalization capabilities across diverse data distributions. In contrast, BERT exhibited significantly poorer performance, with its *F*_1_-score dropping from 0.85 on the test set to 0.48 on the external validation set.

**Figure 3.**
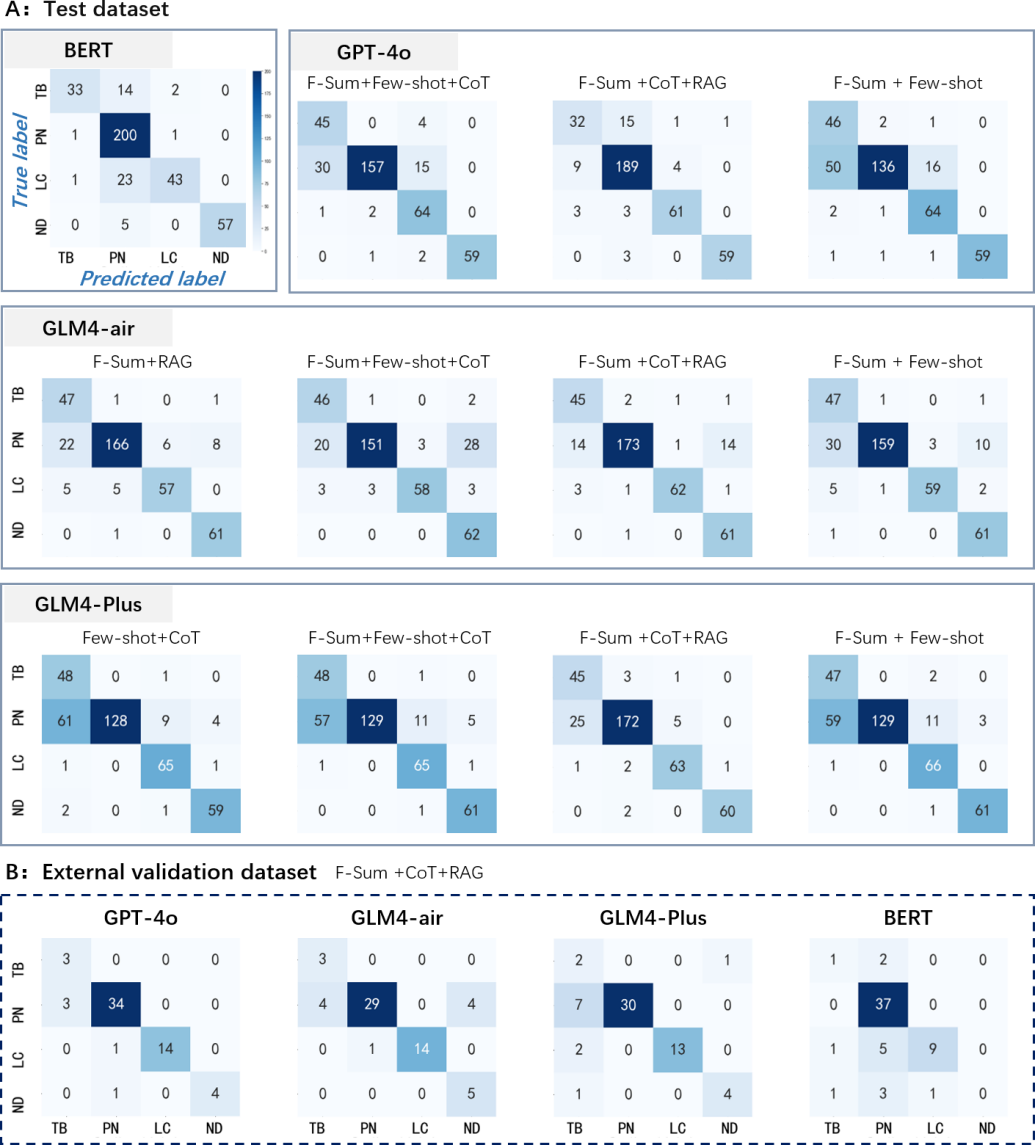
Confusion matrixes of large language model (LLM)–based and Bidirectional Encoder Representations from Transformers (BERT)–based models. CoT: chain of thought; F-Sum: feature summarization; LC: lung cancer; ND: no disease; PN: pneumonia; RAG: retrieval-augmented generation; TB: tuberculosis.

Partial results of the feature summarization have been listed at Table 3 and further analysis of confusion matrices (see Figure 3) revealed nuanced insights into the model performance for specific disease categories. While LLM-based strategies performed well on lung cancer, they were less accurate for pneumonia, likely due to the larger sample size in the training data favoring BERT’s fine-tuned recognition. Using the F-Sum + CoT + RAG method, GPT-4o exhibited low recall (32/49, 65.31%) with 15 cases misclassified as pneumonia (PN) in tuberculosis (TB) prediction, which is similar with the BERT (33/49, 67.35%), while GLM-4-air (recall=42/49, 85.71%) and GLM-4-Plus (45/49, 91.84%) demonstrated significantly superior performance. For PN, GPT-4o (recall=189/202, 93.56%) and BERT (200/202, 99.01%) were the highest, whereas GLM-4-air (173/202, 85.64%) and GLM-4-Plus (172/202, 85.15%) showed notable confusion between PN and TB (14 and 25 misclassifications, respectively). In lung cancer detection, GPT-4o (61/67, 91.04%), GLM-4-air (62/67, 92.54%), and GLM-4-Plus (63/67, 94.03%) surpassed BERT’s recall (43/67, 64.18%), which misclassified 23 lung cancer cases as PN. All models detected healthy individuals well: GPT-4o (60/63, 95.24%), GLM-4-air (61/62, 98.39%), GLM-4-Plus (60/62, 96.77%), and BERT (57/62, 91.94%).

**Table 2.** Performance of large language model (LLM)–based and BERT-based^a^ lung disease prediction models. *F*_1_-score, recall, and precision were all macro averages. The “Few-shot + CoT + RAG (manual prompt)” meant that the samples for few-shot and the questions for chain of thought (CoT) were selected and written by an expert.

Dataset	LLM	*F*_1_-score	Accuracy	Recall	Precision
Test dataset					
F-Sum^b^ + CoT + RAG^c^	GLM-4-air	0.89	0.90	0.92	0.87
F-Sum + RAG	GLM-4-air	0.86	0.87	0.90	0.84
F-Sum + Few-shot + CoT	GLM-4-air	0.83	0.83	0.89	0.81
F-Sum + Few-shot	GLM-4-air	0.85	0.86	0.90	0.83
Few-shot + CoT+ RAG (manual prompt)	GLM-4-air	0.83	0.83	0.86	0.84
F-Sum + CoT + RAG	GLM-4-PLUS	0.89	0.89	0.92	0.87
Few-shot + CoT	GLM-4-PLUS	0.80	0.79	0.88	0.80
F-Sum + Few-shot	GLM-4-PLUS	0.81	0.80	0.89	0.80
F-Sum + Few-shot + CoT	GLM-4-PLUS	0.81	0.80	0.89	0.80
F-Sum + CoT + RAG	GPT4o	0.87	0.90	0.86	0.88
F-Sum + Few-shot	GPT4o	0.81	0.80	0.88	0.80
F-Sum + Few-shot + CoT	GPT4o	0.85	0.86	0.90	0.83
BERT	BERT	0.85	0.88	0.81	0.93
External validation dataset					
F-Sum + CoT + RAG	GPT4o	0.86	0.92	0.91	0.86
F-Sum + CoT + RAG	GLM-4-PLUS	0.72	0.82	0.79	0.74
F-Sum + CoT + RAG	GLM-4-air	0.79	0.85	0.93	0.74
CoT + RAG (manual prompt)	GLM-4-air	0.77	0.85	0.83	0.80
BERT	BERT	0.48	0.78	0.48	0.51

a BERT: Bidirectional Encoder Representations from Transformers.

b F-Sum: feature summarization.

c RAG: retrieval-augmented generation.

**Table 3.** Partial results of the feature summarization.

Feature	Tuberculosis	Lung cancer	Pneumonia	No disease
Calcifications and nodular shadows	Spotty, patchy, or nodular calcifications, predominantly in the apical and posterior segments of the upper lobes, often accompanied by fibrous streaks and pleural traction.	Occasional intralesional calcifications, typically with lobulated margins and spiculations; calcifications are rare in lung cancer.	Calcifications are uncommon; if present, they are scattered and not associated with pleural traction.	No calcifications or abnormal nodules.
Cavity formation	Thick-walled cavities with smooth inner walls, relatively large in diameter, often accompanied by patchy or nodular shadows and surrounding fibrosis.	Rare cavitation; when present, it has irregular wall thickness and surrounding infiltration.	Cavities are rare, observed only in prolonged cases.	No cavitation.

## Discussion

### Principal Findings

LLMs have shown exceptional potential in clinical applications, including disease diagnosis, treatment recommendation, and prognosis prediction. However, challenges such as suboptimal diagnostic accuracy and adherence to clinical guidelines persist. This study addresses these issues by integrating feature summarization, CoT reasoning, and hybrid RAG into a prompt engineering framework, resulting in significant performance improvements in pulmonary disease prediction.

The proposed strategy (F-Sum + CoT + RAG) demonstrated superior performance on both the test dataset (GLM-4-air: *F*_1_-score=0.89, accuracy=0.90; GLM-4-plus: *F*_1_-score=0.89, accuracy=0.89) and the external validation dataset (GPT-4o: *F*_1_-score=0.86, accuracy=0.92). These results highlight the effectiveness of combining feature learning, CoT, and RAG to enhance LLM reasoning and representation capabilities across varying data distributions. Across different LLMs, F-Sum + CoT + RAG consistently outperformed F-Sum + Few-shot + CoT and F-Sum + Few-shot. Notably, GLM-4-Plus achieved the highest *F*_1_-score on the test dataset, while GPT-4o outperformed others on the external validation dataset, achieving the highest *F*_1_-score. The observed performance divergence may arise from the different distributions of diseases in the 2 datasets. On test set, the method with GLM-4-Plus recognizes the TB (45/49, 91.8%) more accurately than the GPT-4o (32/49, 65.31%). However, the accuracy (189/202, 93.6%) of GPT-4o in identifying the PN is higher than the GLM-4-Plus (172/202, 85.15%). For the external dataset, the number of samples classified as the PN (n=37) is far more than the TB (n=3), therefore, the superiority of the GLM-4-Plus in identifying the TB may not be fully reflected in its predictive performance. In general, GPT- or GLM-series LLMs outperform BERT in overall predictive accuracy. However, they exhibit varying predictive performance across different disease categories. Therefore, selecting an appropriate LLM for clinical application may require careful consideration of the primary diagnostic and therapeutic priorities.

Except GLM-series and GPT4-o LLMs, the DeepSeek-V3 (Hangzhou DeepSeek Artificial Intelligence Basic Technology Research Co Ltd) was further tested to validate the generalizability of our method. With the same strategy (F-Sum + CoT + RAG), it achieved 93.16% accuracy on the test set and 86.67% on external validation, which is comparable to GLM or GPT-4o variants. Notably, the result with DeepSeek demonstrated particularly advantage in detecting lung cancer, with an *F*_1_-score of 0.92, which may be attributed to its training focus on oncology literature. Good predictive performance across multiple LLMs (GLM-4, GPT-4o, DeepSeek, etc) confirms the benefits of the proposed method again.

To further evaluate the generalizability of the proposed prompt engineering strategies, we applied the optimal strategy (F-Sum + CoT + RAG) to the external validation dataset. LLMs all demonstrated strong performances, confirming the generalizability of the strategies. In contrast, the BERT model showed a marked decline in generalization capability, with its *F*_1_-score dropping significantly from the test dataset (0.85) to the external validation dataset (0.48). These results underscore the superiority of LLM-based prompt engineering strategies in maintaining performance across multicenter datasets, compared to traditional fine-tuning models.

Feature summarization, a key component of this approach, enables the model to prioritize critical disease characteristics, enhancing its domain-specific knowledge. Compared to the Few-shot + CoT + RAG strategy, feature summarization consistently achieved better results, with an *F*_1_-score of 0.89 versus 0.83 on the test set. On the external validation dataset, Few-shot + CoT + RAG achieved an *F*_1_-score of 0.77, while the feature summarization strategy achieved 0.79. Before K-means clustering, TF-IDF was used to extract important medical words to encode report texts. For comparative analysis, instead of TF-IDF, reports texts were represented with BERT-based embedding vectors. With the GLM4-air, the proposed method (F-Sum with BERT-based embeddings + CoT + RAG) achieved inferior performance (*F*_1_-score=0.86, accuracy=0.86, and precision=0.84) to the method with TF-IDF (*F*_1_-score=0.89, accuracy=0.90, and precision=0.87), which verify the efficiency of the combination of TF-IDF and K-means.

CoT reasoning further improved performance by simulating a clinician’s step-by-step diagnostic process. For example, the inclusion of CoT reasoning raised the *F*_1_-score from 0.86 to 0.8870 for GLM-4-air. RAG enhanced the generative ability of the model by retrieving contextually relevant information, enabling more accurate predictions. Experimental results revealed that multi-path retrieval outperformed single-path retrieval, as it provided more comprehensive background knowledge, enabling the model to make more accurate judgments.

Table S1 in Multimedia Appendix 1 shows that compared to RAGs with either dense or sparse vector representations, the hybrid RAG configuration (dense + sparse) demonstrated obvious superiority, achieving 93.16% accuracy versus 91.32% (sparse) and 88.42% (dense). Using the Shapley value decomposition to quantify 2 representations’ contributions, the dense retrieval is +2.33% and sparse retrieval is +0.88%. The dense vectors capture semantic similarity (eg, “nodular opacities”), while sparse representations ensure keyword matching (eg, “caseating granulomas” for TB).

Despite these advances, certain limitations remain. This method struggled to distinguish between tuberculosis and pneumonia due to overlapping radiological features. For instance, acute miliary tuberculosis may present as diffuse micronodules in both lungs, resembling the findings in viral or *Mycoplasma pneumonia*. While LLMs sacrifice some pneumonia recall in favor of predicting other subcategories, their overall performance remains superior. In contrast, BERT, despite its seemingly good performance in distinguishing between tuberculosis and pneumonia, is highly influenced by factors such as sample imbalance and class size. As a result, BERT tends to overpredict pneumonia due to the higher prevalence of pneumonia cases in the dataset. These challenges illustrate the difficulty of differentiating pneumonia and tuberculosis in certain clinical scenarios. Future studies should integrate richer clinical expertise and multimodal data to address these challenges. Besides, when deploying models in the hospital, to ensure the data security, the model should automatically deidentify patient information. Replacing the existing internet-based application programming interfaces of LLMs with locally deployed models will be necessary to reduce the risk of data leakage.

Based on our newly proposed method, the system can be practically deployed in real-world hospital settings by integrating it into existing hospital information systems and picture archiving and communication systems. Radiology reports of various pulmonary diseases can be continuously added to the RAG-based knowledge base, enabling automatic summarization of disease features and real-time prediction for new reports. In clinical workflows, the system can be triggered after a radiology report is generated, providing diagnostic suggestions to physicians as decision support. The required infrastructure includes connection to EHR and picture archiving and communication systems, a secure backend for model inference, and an interface for physicians to review and interact with AI-generated outputs, thereby enhancing diagnostic accuracy and efficiency.

### Conclusions

In this study, we developed an LLM-based classification model for pulmonary disease diagnosis, leveraging typical disease patterns, simulating diagnostic reasoning processes, and systematically analyzing similar cases. The proposed model achieved significant improvements in diagnostic performance for pulmonary diseases, as validated across multi-center datasets, demonstrating robust generalization capabilities.

By integrating advanced prompt engineering techniques, including feature summarization, CoT reasoning, and RAG, this approach not only enhances predictive accuracy but also lays a foundation for extending auxiliary diagnostic and treatment tasks to other diseases. This work offers valuable insights and a theoretical framework to support the broader application of LLMs in clinical decision-making and medical research.

## Supplementary material

10.2196/72638 Multimedia Appendix 1 BERT-based pulmonary disease classification model and contribution value of dense and sparse retrieval. BERT: Bidirectional Encoder Representations from Transformers.

## Abbreviations

AI: artificial intelligence
BERT: Bidirectional Encoder Representations from Transformers
CoT: chain of thought
CT: computed tomography
EHR: electronic health record
F-Sum: feature summarization
LLM: large language model
NLP: natural language processing
PN: pneumonia
RAG: retrieval-augmented generation
TB: tuberculosis
TF-IDF: term frequency–inverse document frequency
