# Supplementary material for: Enhancing Pulmonary Disease Prediction Using Large Language Models With Feature Summarization and Hybrid Retrieval-Augmented Generation: Multicenter Methodological Study Based on Radiology Report
Source: J Med Internet Res. 2025 Jun 11;27:e72638. doi: 10.2196/72638 (PMC12176309; doi:10.2196/72638)
Supplement: Multimedia Appendix 1 [file jmir-v27-e72638-s001.docx]

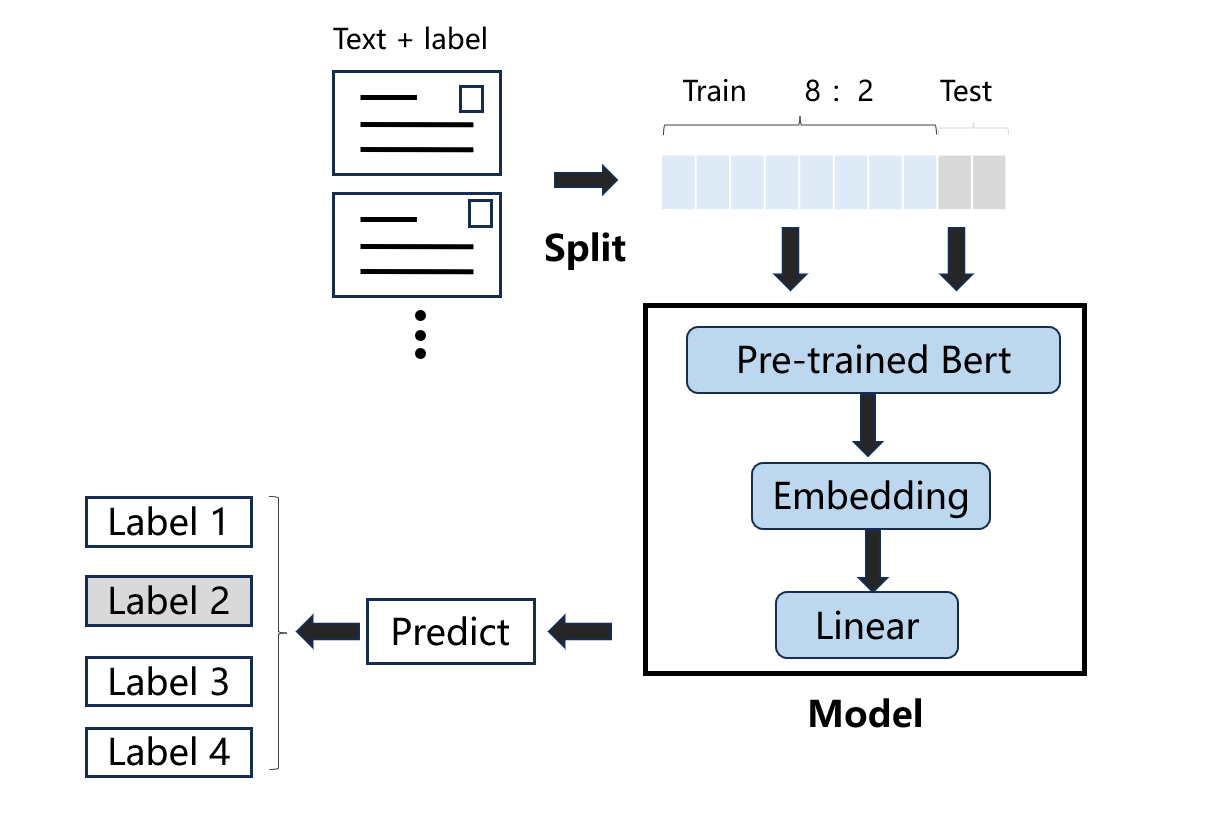


**Figure S1 BERT-based pulmonary disease classification model.**

| Methods | Accuracy | F1 score |
| --- | --- | --- |
| Dense Retrieval | 2.33% | 1.55% |
| Sparse Retrieval | 0.88% | 0.49% |

**Table S1 Contribution value of Dense and Sparse retrieval**
